# Orthokeratology Decentration: Topography Alignment, Tear Profile Symmetry and Induced HOAs

**DOI:** 10.1007/s44402-026-00046-y

**Published:** 2026-03-06

**Authors:** Eihab Eltantawy, Emily Field, Catherine Bui, Michael Kang

**Affiliations:** School of Optometry and Vision Science, University of New South Wales, Sydney, New South Wales Australia

**Keywords:** Chord mu, Higher order aberrations, Orthokeratology, Rotational misalignment, Simulated tear film clearance, Treatment zone decentration

## Abstract

**Purpose:**

To assess how topographic rotational misalignment alters orthokeratology (OK) treatment position, symmetry and visual references. A simulated lens-cornea model that predicts treatment position is introduced. A further aim investigated decentration-induced change in higher-order aberrations, root mean square (ΔHOA RMS).

**Methods:**

Retrospective analysis of topography from 26 myopic OK wearers was conducted on visual and geometric aligned scans. Treatment centres were obtained from tangential maps using ellipse fitting, while chord mu was measured from visual scans. MATLAB modelling related Placido misalignment with treatment position and used lens-cornea height data to generate tear film profiles. An optimisation protocol then identified lens shift and tilt that minimised clearance asymmetry. Lastly, Zernike fits across various pupil sizes, enabled stepwise ΔHOA RMS calculation for decentrations up to 1.0 mm.

**Results:**

Rotational misalignment was strongly associated with a change in treatment position (*r* = −0.93, *p* < 0.001) and geometric modelling closely matched measured shifts (*r* = 0.98, *p* < 0.001). Corneal symmetry indices did not differ between visual and geometric scans and chord mu remained stable after treatment (mean change −0.006 mm, *p* = 0.67). The tear film model predicted horizontal decentration well (*r* = 0.77, *p* < 0.001; mean absolute error = 0.13 mm; bias = −0.02 mm, limits of agreement −0.36 to 0.31 mm), whereas vertical prediction was weak (*r* = 0.23, *p* = 0.27). Treatment showed a temporal bias on visual scans that diminished after geometric recentring. Simulated ΔHOA RMS increased with decentration, for a baseline value of 0.10 µm and a 4.0 mm pupil, ~0.40 mm decentration would double RMS to ~0.20 µm.

**Conclusions:**

Visual scan alignment provides stable references for OK centration without distorting treatment appearance. Integrating topography with lens parameters through tear film symmetry modelling may help predict decentration, guiding lens design to optimise visual outcomes.

Key Points

- Rotational misalignment does not distort orthokeratology treatment position or symmetry, and instructing subjects to fixate coaxially during capture ensures reference points are visually meaningful.
- A tear film MATLAB model that combines corneal elevation with lens sagittal height can predict horizontal orthokeratology treatment decentration with good agreement to clinical measurements; however, weak vertical prediction implicates non-corneal factors such as eyelid forces.
- Simulated decentration shows a nonlinear rise in higher-order aberrations. For a 4.0 mm pupil and a typical baseline value of 0.10 µm, root mean square higher-order aberrations approximately double at ~0.40 mm decentration, providing a clinically useful heuristic threshold for balancing myopia control benefit against visual quality.

## Introduction

Orthokeratology (OK) is a reversible, overnight gas permeable (GP) contact lens therapy that temporarily reshapes the anterior cornea to provide clear daytime vision [1]. The treatment produces a central zone of relative corneal flattening surrounded by a paracentral steepened annulus. OK is now widely used for paediatric myopia management and can slow axial elongation. This has primarily been attributed to induced peripheral myopic defocus and, to a lesser extent, the effects of spherical aberration [2]. Therefore, OK treatment often pursues one of two aims depending on the intended application: (1) full refractive correction with minimal degradation of image quality and (2) enhancement of the myopia control signal through greater peripheral defocus. OK is becoming increasingly customisable beyond targeted refractive correction by adjusting lens parameters such as back optic zone diameter and asphericity/eccentricity to modulate higher-order aberrations (HOAs) [2–4]. Achieving the intended optical effect depends on where the treatment zone ultimately localises on the cornea. Accordingly, accurate prediction of overnight lens position is important for anticipating both treatment location and the likely impact on vision. Poor centration degrades image quality due to induced coma [5]. This is associated with symptoms such as ghosting, glare and monocular diplopia. However, mild amounts of coma may contribute modestly to myopia control by enhancing the peripheral defocus effects of OK [2]. If the extent of treatment decentration and resulting induced HOAs can be predicted, then these aberrations may be better leveraged to maximise treatment efficacy.

Most studies report a characteristic inferotemporal bias in OK treatment position, commonly attributed to corneal asymmetry and eyelid forces [5–7]. Gravity can also influence GP lens positioning [8, 9], but its role in OK has not yet been examined in sufficient detail. To evaluate the OK treatment location, a reference must be chosen by the practitioner. Either the pupil centre or the corneal vertex is used to evaluate the magnitude of decentration [5, 10]. Ultimately, the clinical relevance of these landmarks is influenced by the manner in which a topography scan is captured. In a visual captured scan, a subject fixates accurately along the axis of the instrument. This will cause the cornea to appear tilted with respect to the topographer as the eye rotates to account for the temporal position of the fovea. In a Placido-based system, the rings will appear shifted towards the nasal limbus. This rotational misalignment is expected and visually meaningful for two reasons. Firstly, the pupil centre coordinates become equivalent to the corneal sighting centre (CSC), i.e., the corneal intercept representing the line of sight. Secondly, the corneal vertex, represented as the topographic origin, becomes the coaxially sighted corneal light reflex (CSCLR). The linear distance between these two reference points is referred to as chord mu [11]. To correct the rotational misalignment during visual capture, some practitioners recentre corneal topography with reference to the horizontal limbal boundaries. This is referred to as geometric capture and is a reasonable approximation to apex alignment methods described in the literature [12]. The rationale is that this produces maps that more faithfully represent corneal shape, thereby improving lens design and interpretation of treatment effect. Reducing asymmetry introduced by ocular rotation may also be advantageous when screening for corneal irregularity due to pathology. This approach is informed by studies showing that rotational misalignment can artefactually create pseudo-keratoconus patterns on topography [13, 14]. However, geometric scans divorce the data from visually relevant references needed to guide clinical decisions. One of the aims of this study is to investigate the effect of geometric captured scans on the evaluation of OK treatment position and symmetry.

In most myopic eyes, the CSCLR is the closest practical surrogate for the visual axis corneal intercept [15–17]. If the aim of OK is optimal visual quality, then treatment must be centred on visually meaningful landmarks and lens designs should aim to promote this. In modern refractive surgery, this is achieved by centring at the CSCLR, the CSC or at a point between them [18, 19]. The stability of these landmarks is also an important consideration. The CSC is the corneal projection of the pupil centre, which is susceptible to vary dynamically to changing illumination levels [20]. This may affect referencing the OK treatment position, relative to the pupil, between visits. Additionally, OK alters corneal shape and thus the position of these landmarks is also susceptible to change. This has implications for generating treatment difference maps, whereby anomalous corneal points are used for subtraction. A further aim of this study is to examine the stability of these reference points by comparing chord mu before and after OK treatment.

The location of visually significant reference points must be considered by practitioners when designing lenses and initiating treatment. However, accurately predicting the centration of OK treatment and the effect on visual quality remains a practical challenge. Most models that predict OK decentration rely primarily on corneal morphology and ignore lens design entirely [7, 21–27]. This omission is counterintuitive, as lens parameters are selected specifically to optimise centration. Furthermore, in these studies, peripheral corneal data frequently demonstrate superior predictive accuracy than central metrics. Parameters often assessed include toricity, eccentricity and elevation asymmetry measured at fixed chords, often near 8 mm. However, the accuracy of peripheral corneal data is influenced by factors such as incomplete scan coverage, eyelid/eyelash artefacts, tear film disruption, interpolation and extrapolation errors [28]. A novel strategy introduced in this study considers the entire dynamic tear film profile created by the interaction between an OK lens and the cornea. This strategy aims to optimise the overall symmetry of the profile, as the lens ‘seeks’ a mechanically balanced position on the eye. Topography-derived measures of corneal shape and symmetry can therefore become integrated with customised lens design to improve the prediction of OK lens position. These predictions can inform parameter changes that steer the lens to the most visually optimal landmark and improve treatment outcomes.

In summary, this study aims to achieve the following: (1) assess the effect of topographic rotational misalignment on OK treatment interpretation by comparing visual and geometric scans, (2) examine the stability of chord mu before and after OK, (3) evaluate a simulated lens positioning framework that attempts to optimise tear film profile symmetry and predict OK treatment decentration, (4) benchmark observed treatment locations against the literature-reported inferotemporal tendency and (5) model the HOAs induced by OK decentration to examine the effect on visual quality. This could provide clinicians with a clearer, reproducible foundation for lens design and treatment evaluation.

## Materials and Methods

### Subjects

This study was a retrospective analysis of pre- and post-treatment OK corneal topography data. The research was performed in accordance with the principles of the Declaration of Helsinki and its later amendments (or comparable ethical standards). Ethical approval was obtained from the Human Research Ethics Advisory Panel, University of New South Wales, Australia (approval number iRECS8446). A total of 26 patients undergoing OK treatment for myopia were included in the study. Corneal topography scans were obtained using the Medmont E300 corneal topographer (medmont.com.au) with the Medmont Studio 7 software. Both geometric and visual aligned scans were required at baseline and post-treatment (considered as a minimum of 1 month after initiation of OK). Only scans of sufficient quality were eligible (i.e., no significant Placido tear film distortion or ring defocus, minimal eyelid/eyelash artefact and good corneal coverage with clear pupil delineation). As this was a retrospective dataset, multiple eligible captures per condition and time point were not consistently available. Therefore, averaging across multiple scans was not performed to avoid introducing artefactual variability from lower-quality data. When multiple eligible scans were present, the scan with the highest quality was selected through visual inspection by the same experienced observer. Subjects with corneal disease, prior refractive surgery or substantial corneal toricity were excluded. Toricity was defined from baseline topography as the absolute sagittal height difference at the 8 mm chord between the steep and flat principal meridians (as defined by the Medmont software). Eyes were excluded if this difference exceeded 30 µm on either visual or geometric scans. Participants were fitted with Eyespace (Forge, eye.space) OK spherical lenses, and all were included in the statistical analyses. Table 1 outlines the participant characteristics and lens design parameters used. A priori power analysis (G*Power v3.1.9.6, psychologie.hhu.de/arbeitsgruppen/allgemeine-psychologie-und-arbeitspsychologie/gpower) indicated that detecting a large effect (Cohen’s *d* = 0.80) with 80% power at *α* = 0.05 required a minimum of 15 participants. Additionally, the chosen sample size of 26 was consistent with previous studies investigating OK treatment position [29–31].

**Table 1:** Participant characteristics and orthokeratology (OK) lens parameters.

Variable	Value
Age (years)	23 ± 11 (10–59)
Myopia, sphere (D)	−3.22 ± 1.63 (−1.00 to −8.00)
Refractive astigmatism, cylinder (D)	−0.30 ± 0.32 (0.00 to −0.75)
Back optic zone radius, BOZR (mm)	8.71 ± 0.47 (8.00–9.60)
Sagittal height at 9 mm (µm)	1389 ± 38 (1320–1475)
Lens material	Boston XO: 26 (100%)
Overall diameter (mm)	10.78 ± 0.26 (10.30–11.20)
Back optic zone eccentricity, BOZe	0.00 ± 0.00
Back optic zone diameter, BOZD (mm)	5.70 ± 0.42 (5.00–6.50)
Treatment zone diameter, TZD (mm)	7.77 ± 0.50 (6.30–8.00)
Landing zone angle, LZA (°)	32.7 ± 1.1 (30.8–34.8)
Landing zone eccentricity, LZe	0.74 ± 0.37 (0.00–1.20)

Continuous variables are presented as mean ± standard deviation (range). Categorical variables are presented as number (percentage).

### Corneal Topography

**Fig. 1:**
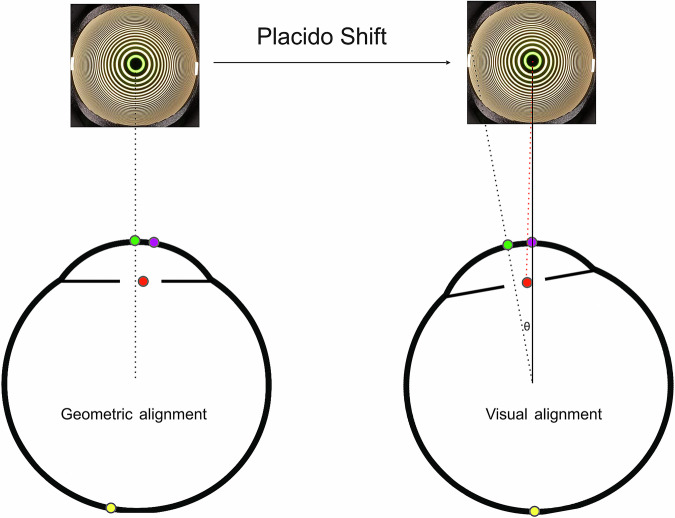
Placido alignment with respect to fixation. In the first position, the participant is asked to fixate nasally from the centre of the Placido rings to achieve geometric centring of the rings with respect to the corneal boundaries. The position of the coloured markers represents the following: green, original vertex normal position, purple, treatment centre, red, pupil centre and yellow, fovea. In the second position, the participant is asked to fixate directly in the centre of the Placido rings. This results in a shift of the Placido ring reflections on the cornea as the eye rotates by angle *θ*. In this example, the treatment centre is approximately coincident with the ‘new’ vertex normal position and is equivalent to the subject fixated coaxial light reflex.

All topography scans used in the study were captured using the same Medmont E300 topographer. As shown in Fig. 1, visual scans were taken by instructing the subject to fixate accurately along the instrument at the very centre of the Placido rings. Geometric scans were taken by asking the subject to change their fixation from the direct centre of the rings nasally to the immediately adjacent rings in gradual steps. This enabled the practitioner to ensure that the Placido reflection was centred with respect to the horizontal limbal boundaries in the capture real-time preview. Both sets of scans were taken prior to commencing OK, and consequently, after the establishment of treatment. Topography scans were acquired in the same room under mesopic illumination. Overhead lights were switched off, and ambient light was minimised by closing the door. Illumination was limited to the Medmont E300 intrinsic light source and the acquisition monitor. The raw topography data from corneal height and tangential curvature topography scans were later imported into MATLAB R2025a (mathworks.com) for analysis using a series of custom scripts and applications.

### Treatment Zone Decentration and Symmetry

A custom MATLAB script was used to generate tangential curvature post-treatment colour maps from both the geometric and visual scan raw data. The generated maps were inspected to identify the characteristic OK treatment pattern, i.e., central flattening with adjacent paracentral steepening typical of myopic OK. Manual filtering was then performed by a single observer for all scans to eliminate peripheral data lying beyond the area of paracentral steepening. This effectively isolated the paracentral zone and eliminated erroneous data, which could skew the calculation of the treatment location. Following manual filtering, the algorithm isolated the treatment zone by identifying the point of maximum corneal curvature radially every 1.2°, in line with the radial construction of the raw topography data. The treatment zone was delineated according to the best-fit ellipse of points of maximum corneal curvature circumferentially along these radial lines. The Cartesian coordinates of the best-fit ellipse centroid defined the treatment position relative to the topographic origin of the scan. This is analogous to the method used by Sun et al. [5]. The change in horizontal pupil position between geometric and visual scans was defined as the amount of linear Placido shift. The extent of the Placido shift was assessed against the change in treatment location relative to the pupil centre between the two scans.

The coordinates of the generated treatment centre relative to the corneal vertex were then used as the point of origin for the evaluation of treatment symmetry. The purpose was to determine if the appearance of OK treatment was significantly different between geometric and visual scans. Medmont style symmetry indices were then calculated for both sets of scans. Data points within a 3 mm radius of the treatment centre were included in the symmetry calculations. This included a left-right (LR) index, analogous to the Medmont inferior-superior index, but across the opposing midline (i.e., the mean difference in corneal power between the left and right hemispheres within the same analysis zone). The surface asymmetry index (SAI) compares diametrically opposite semi-meridians, and lastly, a surface regularity index (SRI). This is a centrally weighted regularity measure where the deviation of each data point’s power from the local neighbouring points is computed and averaged.

### Chord Mu

For the purposes of this study, chord mu was considered as the absolute horizontal linear separation of the pupil centre and topographic origin (i.e., corneal vertex) on the visual scans. During capture, the Medmont software delineates the boundaries of the pupil using edge detection and defines its centre coordinates. The *x* coordinate of the pupil centre in the visual scans is thus equivalent to chord mu. These values at baseline and post-treatment were taken from the visual scans to evaluate the consistency of the corneal vertex position.

### Rotational Misalignment Between Geometric and Visual Scans

Raw corneal elevation data from geometrically aligned scans were imported into a custom MATLAB application and interpolated to generate a smooth height map. The treatment centre coordinates (xT, yT) were plotted on the surface to obtain the corresponding sagittal height (zT). The corneal surface was then rotated vertically and/or horizontally as required, and the topographic origin, i.e., corneal vertex, was recalculated after each rotation (defined as the point of maximum elevation). The treatment centre location was updated according to the applied rotation relative to the new vertex. Equivalent treatment coordinates from the patient’s visual scan were used as the target for a two-stage MATLAB optimisation procedure consisting of an initial pattern search followed by fmincon (MATLAB’s constrained nonlinear optimiser). In this context, pattern search evaluates a small mesh of candidate rotations and steps to any point that reduces the mismatch between the modelled and target treatment position. Fmincon then uses a gradient approach to refine the solution further. The objective was to estimate the corneal rotation (*θ*) that maps the initial geometric treatment centre coordinates to the target visual treatment centre values. The pupil centre position was then mapped relative to this solution, and the coordinates (xP, yP, zP) were computed. Specifically, zP was estimated by considering the change in treatment position relative to the pupil between the two scans (ΔxTP). The effect of corneal rotation on the horizontal position of the treatment, relative to the pupil centre, could then be related mathematically using Eq. 1:

1 $$\Delta {{{\rm{xTP}}}}({{{\rm{\theta }}}})=(\cos {{{\rm{\theta }}}}-1)({{{\rm{xT}}}}-{{{\rm{xP}}}})+\sin {{{\rm{\theta }}}}({{{\rm{zT}}}}-{{{\rm{zP}}}})$$

Where:

*θ*: corneal rotation angle (in radians) about the *y* axis;

xT: treatment centre horizontal coordinate (mm) in geometric scan;

xP: pupil centre horizontal coordinate (mm) in geometric scan;

zT: treatment centre depth coordinate (mm) in geometric scan;

zP: sagittal depth of the pupil, i.e., external anterior chamber depth (mm);

ΔxTP(*θ*): change in treatment horizontal position relative to the pupil between geometric and visual scans (mm).

The first term (cos*θ*−1) (xT−xP) accounts for the cosine foreshortening of any pre-existing horizontal offset between the treatment and pupil when the eye rotates by angle *θ*. The second term, sin*θ* (zT−zP), reflects the parallax effect, i.e., the depth difference between the two points projected onto the horizontal axis under rotation. To validate this model, a fixed zP value of 3.61 mm was used to represent a typical external anterior chamber depth value for a myopic subject [32]. The remaining variables were obtained from the MATLAB application. As a result, the projected change in treatment location relative to the pupil could be assessed against the actual measured change from the raw geometric and visual topography scan data.

### Prediction of OK Decentration

Corneal elevation and lens sagittal height data from post-treatment geometric and visual scans were imported into a custom MATLAB application to generate a simulated tear film clearance map, with the lens centre defined as the origin. Clearance was analysed in the raw Medmont sampling geometry (300 meridians at 1.2° intervals). The clearance map was partitioned into four 90° sectors (±45° about the horizontal and vertical axes, 75 meridians per sector). Within each sector, each meridian was spline interpolated onto a common radial vector spanning 0–6 mm (601 samples, 0.01 mm steps) and the mean clearance at each radius was computed to form sector-averaged profiles. LR sector averages were used to produce a horizontal tear film clearance profile, while up-down sector averages produced the vertical profile.

From the topographic origin, the lens surface was allowed to translate and tilt about the *x* and *y* axes to emulate in vivo behaviour. Translation was bounded to ±2.0 mm in both the horizontal and vertical directions, and tilt was bounded to ±10° about each corresponding axis. The initial lens position used was consistent for all participants (i.e., no lens translation or tilt). Lens movements updated the clearance map and profiles in real time. Clearance was governed by Eq. 2:

2 $$c(r)={z}_{{{\mathrm{lens}}}}(r)-{z}_{{{\mathrm{cornea}}}}(r)$$

Where:

*r*: radius along the common axis relative to the current lens centre;

*c*(*r*): the clearance (tear film thickness) evaluated at radius *r*;

*z*_lens_(*r*): sagittal height (depth coordinate) of the lens back surface evaluated at radius *r*;

*z*_cornea_(*r*): sagittal height (depth coordinate) of the cornea evaluated at radius *r*.

To predict the natural lens resting position, a two-stage MATLAB optimisation procedure, analogous to that described earlier, was used to identify the combination of lens translation and tilt which minimised asymmetry of the horizontal and vertical tear film profiles. Clearance values for mirrored quadrants were paired at matched distances from the centre, and asymmetry was quantified from the clearance profile. Specifically, this was defined as the sum of squared paired differences using Eq. 3:

3 $$A={\sum }_{k=1}^{n}{[c(-rk)-c(rk)]}^{2}$$

Where:

*A*: the objective asymmetry function to be minimised;

*c*(*rk*): clearance at the *k*th paired sampling distance from the lens centre.

**Fig. 2:**
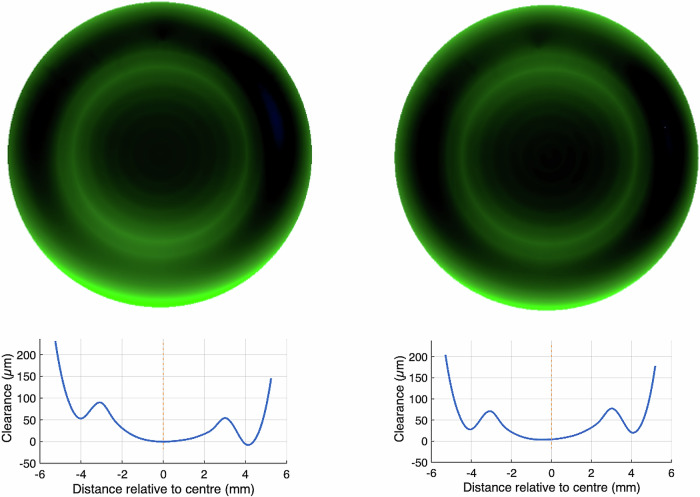
Predicted lens treatment position determined through optimisation of tear film profile symmetry. Simulated fluorescein patterns (top figures) and vertical average tear film profiles, before (lower left) and after (lower right) optimisation is shown.

This was evaluated in 0.01 mm steps over the region common to both the corneal profile and the lens footprint (i.e., radii up to the maximum available lens radius). The effect of this is shown in Fig. 2. The resulting horizontal and vertical lens translations were taken as the predicted OK treatment decentration amounts and were compared with the previously measured treatment coordinates.

### Simulated Effect of Lens Decentration on HOAs

Raw corneal elevation data from both visual and geometric aligned scans were imported into MATLAB. A circular 4.0 mm pupil region was initially centred on the treatment zone (i.e., the ellipse fit treatment centroid from the post-treatment tangential map) to analyse HOAs. Within this region, the corneal surface was fitted by least squares using the Optical Society of America and the American National Standards Institute standardised Zernike polynomials. The resulting elevation coefficients were converted to single surface optical path differences (OPD, µm). Higher order OPD terms were then combined in quadrature to compute the HOAs root mean square (HOA RMS, µm). To assess the sensitivity of HOAs to treatment decentration, the pupil centre was displaced over a polar grid from 0 to 1.00 mm in 0.10 mm steps at 30° intervals. For each offset, the Zernike coefficients and HOA RMS were recomputed. Heat maps were generated to visualise the change relative to baseline. Individual maps were then combined to produce a global mean map, from which directional averages were derived.

### Statistical Analysis

All statistical analyses were conducted in MATLAB R2025a (mathworks.com). Absolute chord mu values were measured from visual scans. For pre- and post-treatment data, the mean and standard deviation were calculated. Normality was assessed using the Lilliefors test [33] and paired *t*-tests evaluated mean change. Effect size was reported as Cohen’s *d*. Pearson correlation was used to assess the association between the change in treatment position relative to the pupil centre (ΔxTP) and the extent of Placido shifting. Symmetry indices (LR, SRI, SAI) from the visual and geometric scans were compared with paired *t*-tests. The mean difference between the mathematically predicted and measured ΔxTP was evaluated with a paired *t*-test. Agreement between predicted and measured values was examined further with Pearson correlation. Absolute prediction error was summarised by the mean absolute error (MAE). Also, Pearson correlation was used to assess the association between MATLAB-predicted and clinically measured treatment shifts in the horizontal and vertical directions. Predictive accuracy was evaluated with Deming regression analysis [34], and absolute prediction error was summarised using the MAE. Agreement was assessed with Bland–Altman analysis. For simulated decentration magnitude, the mean and standard deviation of the induced HOAs (ΔHOA RMS) were calculated. Slopes (µm/mm) were then determined to characterise the rate of change in mean ΔHOA RMS with increasing decentration.

### Ethical Approval

This study was performed in accordance with the principles of the Declaration of Helsinki and its later amendments (or comparable ethical standards). Ethical approval was granted by the Human Research Ethics Advisory Panel (HREAP), University of New South Wales, Australia (approval number iRECS8446).

## Results

### The Effect of Placido Rotational Misalignment on Treatment Position and Symmetry

**Fig. 3:**
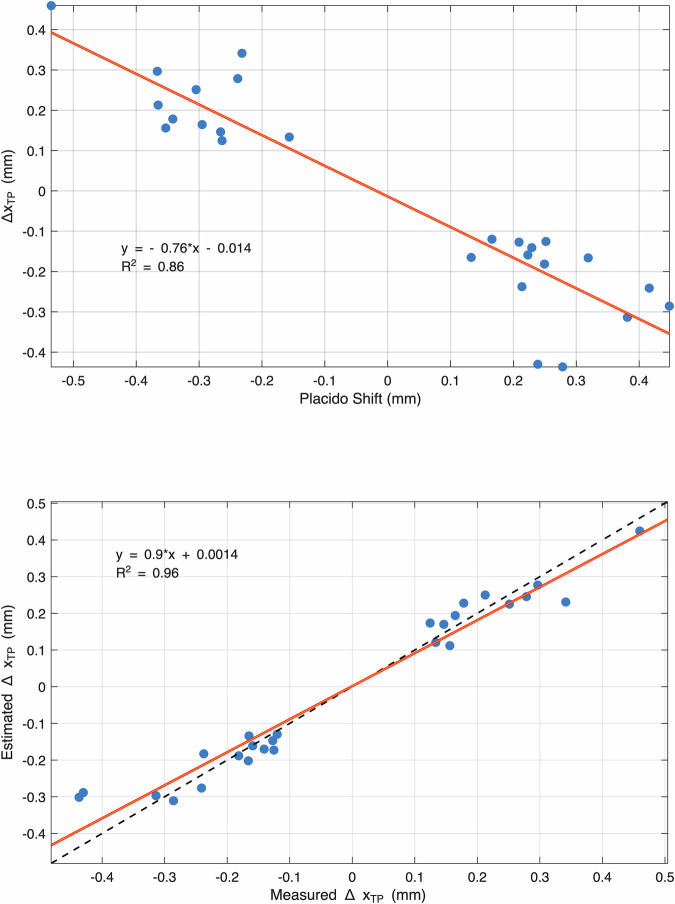
Evaluation of treatment centre position (ΔxTP) relative to the pupil centre. Linear regression lines are shown in both plots. The top figure shows an inverse correlation between the Placido shift and the change in treatment position with respect to the pupil centre. The bottom figure compares the measured and estimated shift in treatment position. The red regression line is shown with the *x*, *y* identity equivalence black dotted line. The respective regression equations and *R*^2^ values are also shown.

Figure 3 shows the relationship between the change in treatment position relative to the pupil centre (ΔxTP) and the extent of Placido shift. This was examined to determine the influence of rotational misalignment on treatment position. Pearson correlation showed a strong negative association (*r* = −0.93, *p* < 0.001), indicating that Placido shift was accompanied by oppositely directed displacement in treatment position.

Predicted ΔxTP values from Eq. 1 (substituting a constant value for zP of 3.61 mm) were compared with the measured ΔxTP using a paired *t*-test. The mean paired difference (predicted − measured) was 0.003 mm (SD: 0.055, 95% CI: −0.019 to 0.025). There was no evidence of a significant mean difference: *t* = 0.27, *p* = 0.79, MAE 0.041 mm. A strong positive correlation between predicted and measured values can also be seen in Fig. 3 (*r* = 0.98, *p* < 0.001). This supports the model’s high predictive accuracy with minimal bias. The flattening relative to the identity line in the regression plot indicates underestimation at extreme values, consistent with using a cohort-average zP that understates shifts in eyes with deep anterior chambers.

Corneal symmetry indices (LR, SRI, SAI) were compared between the visual and geometric scans using paired *t*-tests to assess the effect of rotational misalignment when metrics were referenced to the treatment centre. As summarised in Table 2, there was no evidence of systematic differences between visual and geometric centring across indices. LR: mean difference = −0.014, 95% CI = −0.218 to 0.189; *t *= −0.14, *p* = 0.89. SAI: mean difference = −0.065, 95% CI = −0.224 to 0.094; *t* = −0.84, *p* = 0.41. SRI: mean difference = 0.008, 95% CI = −0.080 to 0.095; *t* = 0.18, *p* = 0.86. This suggests that symmetry metrics remain stable despite ocular rotation when measurements are taken with respect to the treatment centre.

**Table 2:** Symmetry indices: visual (V) vs geometric (G) scans.

Symmetry index	Mean *V*	Mean *G*	Mean difference (95% CI)	*t*	*p* value
LR tangential	0.058	0.043	−0.014 (−0.218 to 0.189)	−0.14	0.89
SAI tangential	2.263	2.198	−0.065 (−0.224 to 0.094)	−0.84	0.41
SRI tangential	1.109	1.117	0.008 (−0.080 to 0.095)	0.18	0.86

Mean difference is Mean *V* − Mean *G* (95% CI). *t*, *p* from paired *t*-test.

*LR* left-right corneal symmetry index, *SAI* surface asymmetry index, *SRI* surface regularity index.

### Stability of Chord Mu

**Fig. 4:**
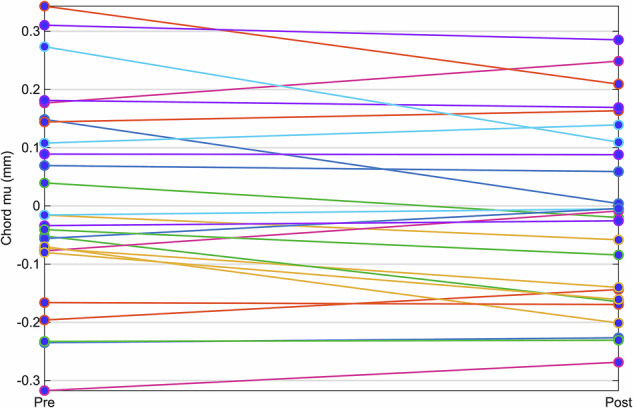
Individual paired changes in chord mu from pre- to post-treatment. The ‘spaghetti’ plot shows within-eye trajectories of chord mu at two time points (pre, post). Each line connects the paired measurements from a single eye, with circular markers at each time point. The *y*-axis denotes chord mu.

Horizontal absolute chord mu values measured from the visual scans were compared before and after OK treatment. This was determined as the absolute pupil *x* coordinate relative to the corneal vertex. Figure 4 shows the within-subject changes. Paired differences were approximately normal (Lilliefors *p* = 0.34). The pretreatment mean was 0.136 mm (SD: 0.099), and the post-treatment mean was 0.130 mm (SD: 0.088). The mean paired difference (post − pre) was −0.006 mm (SD: 0.072 mm), 95% CI: −0.035 to 0.023. A paired *t*-test indicated no evidence of change, *t *= −0.43, *p* = 0.67 and the effect size was negligible (Cohen’s *d* = −0.08). Overall, chord mu decreased by about 6 µm on average, indicating no significant systematic shift. This reflects the relative stability of the pupil centre and corneal vertex after treatment.

### Prediction of OK Lens Decentration

**Fig. 5:**
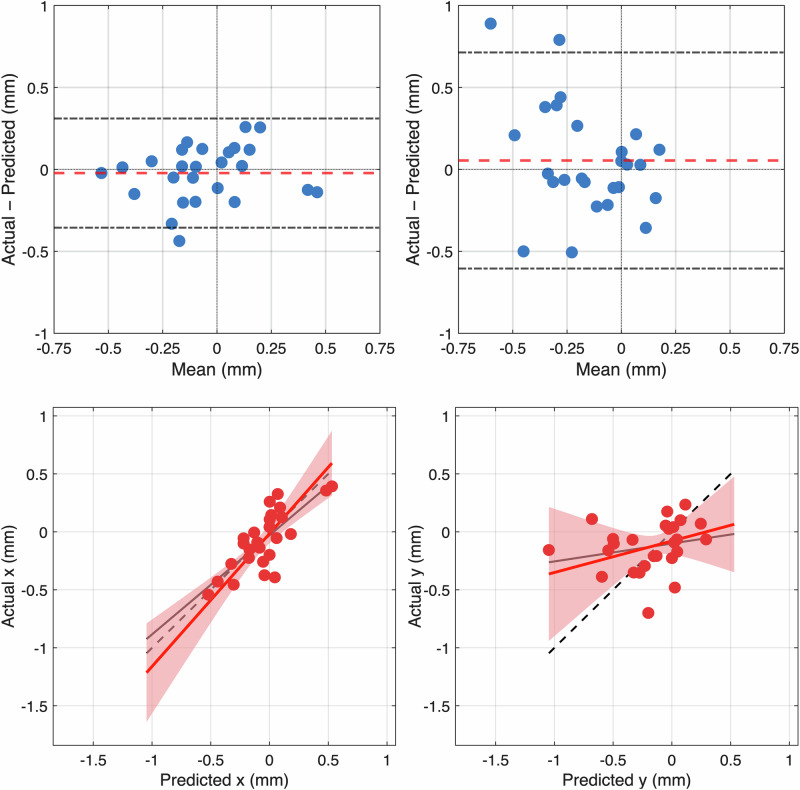
Analysis of predicted vs actual treatment position. Top figures show Bland–Altman plots for *x* and *y* treatment position, dotted lines indicate bias and the limits of agreement. Bottom figures show Deming regression results. Deming line with 95% confidence intervals (red), identity line (dotted black) and ordinary least squares line (solid black).

Figure 5 summarises the predictive performance of the MATLAB OK decentration model against clinical measurements. For horizontal decentration, predicted and measured values were strongly correlated (*r* = 0.77, *p* < 0.001). The error was modest (MAE 0.133 mm). Bland–Altman analysis indicated negligible bias, −0.022 mm with 95% limits of agreement (LoA) −0.355 to 0.311 mm. Deming regression estimated a slope of 1.146 (95% CI: 0.709–1.582) and an intercept of −0.016 mm (95% CI: −0.099 to 0.068). Excluding optic zone data reduced performance (*r* = 0.55, *p* = 0.004). Overall, horizontal predictions showed good concordance. In the vertical meridian, the association was weak (*r* = 0.23, *p* = 0.27) and the error was larger (MAE 0.247 mm). Bland–Altman bias was 0.054 mm with LoA −0.605 to 0.714 mm. Deming regression estimated a shallow slope of 0.272 (95% CI: −0.347 to 0.891) and an intercept of −0.079 mm (95% CI: −0.194 to 0.036), indicating poor vertical prediction.

**Fig. 6:**
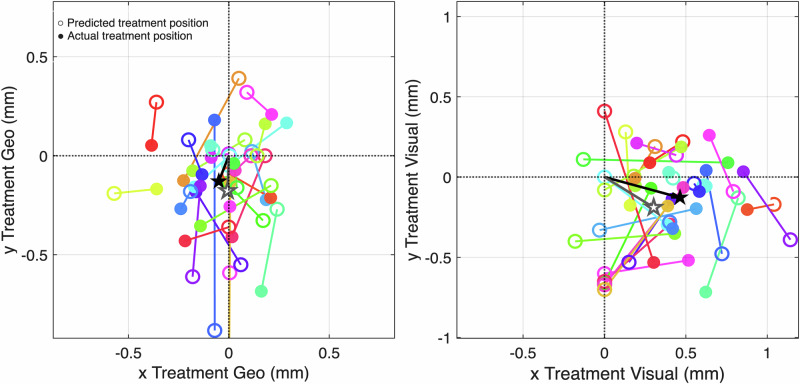
Predicted and actual treatment position for both geometric (Geo) and visual scans. Open circles denote predicted treatment location and closed circles denote actual treatment location. Paired connector lines are shown with black stars denoting mean vector positions. Positive *x* and *y* values represent temporal and superior positions, respectively.

Figure 6 plots the predicted and measured decentration for both visual and geometric scans. With respect to the CSCLR, decentration shows a temporal bias. In geometric scans, this temporal bias diminished, and only a slight inferior bias is observed.

### Simulated Effect of Lens Decentration on HOAs

**Fig. 7:**
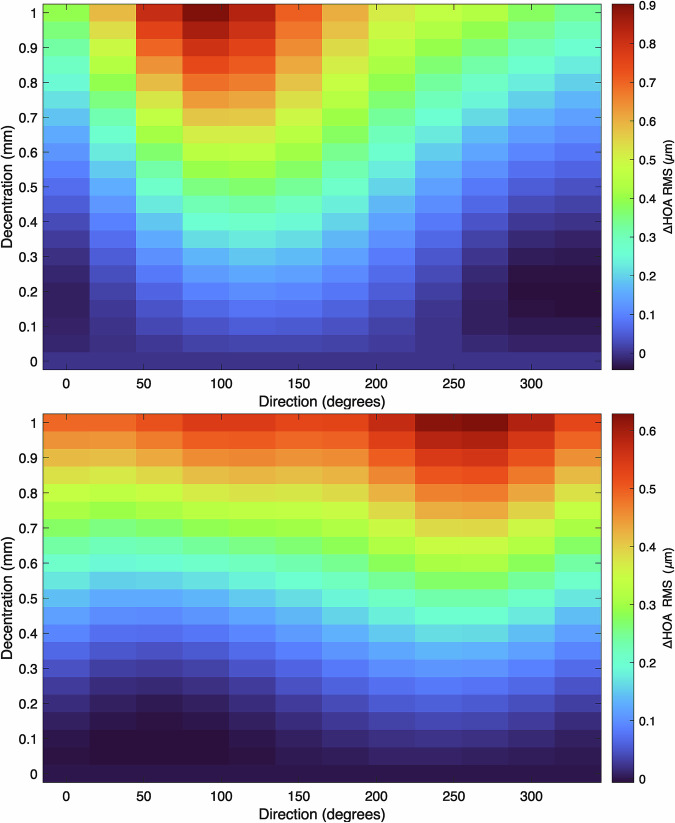
Heatmaps of induced higher-order aberrations root mean square (HOA RMS) for a 4.0 mm pupil as a function of simulated treatment decentration amount and direction. The top map represents data from an individual patient scan, bottom map represents an average of all scans.

Figure 7 shows ΔHOA RMS increasing with simulated treatment decentration for a 4.0 mm pupil. Directional dependence was evident in individual heat maps and reduced in the across-subject average map. Table 3 summarises the mean ΔHOA RMS across various pupil sizes, computed at 12 directions (30° steps) for each decentration band and then averaged. Mean ΔHOA RMS increased with decentration and accelerated with larger offsets. The effect was proportional to pupil size. Additionally, directional variability also increased with decentration and pupil size. For a 4.0 mm pupil, mean ΔHOA RMS slopes (µm/mm) by decentration band were 0.19 (0–0.25 mm), 0.51 (0.25–0.50 mm), 0.71 (0.50–0.75 mm) and 0.77 (0.75–1.00 mm). Assuming a baseline HOA RMS of 0.10 µm (a typical value for normal eyes and a 4.0 mm pupil) [35], an approximate doubling to 0.20 µm occurred at ~0.40 mm decentration; this value is heuristic and will vary with pupil size and baseline aberrations.

**Table 3:** Mean induced higher-order aberrations root mean square (HOA RMS) versus decentration.

Decentration (mm)	3.0 mm ΔHOA RMS (µm)	4.0 mm ΔHOA RMS (µm)	5.0 mm ΔHOA RMS (µm)	6.0 mm ΔHOA RMS (µm)
0.0	0.000 ± 0.000	0.000 ± 0.000	0.000 ± 0.000	0.000 ± 0.000
0.1	0.002 ± 0.005	0.008 ± 0.011	0.017 ± 0.017	0.018 ± 0.024
0.2	0.010 ± 0.010	0.030 ± 0.020	0.066 ± 0.033	0.071 ± 0.047
0.3	0.022 ± 0.014	0.067 ± 0.029	0.141 ± 0.048	0.152 ± 0.069
0.4	0.038 ± 0.017	0.116 ± 0.035	0.235 ± 0.059	0.256 ± 0.088
0.5	0.059 ± 0.019	0.175 ± 0.040	0.340 ± 0.068	0.373 ± 0.105
0.6	0.083 ± 0.019	0.242 ± 0.043	0.452 ± 0.075	0.500 ± 0.118
0.7	0.111 ± 0.019	0.315 ± 0.045	0.564 ± 0.079	0.634 ± 0.128
0.8	0.142 ± 0.018	0.392 ± 0.046	0.674 ± 0.082	0.768 ± 0.137
0.9	0.177 ± 0.018	0.470 ± 0.047	0.777 ± 0.084	0.903 ± 0.145
1.0	0.214 ± 0.018	0.547 ± 0.047	0.872 ± 0.086	1.039 ± 0.155

ΔHOA RMS indicates the change in HOA RMS across 3–6 mm pupil diameters. Values are the mean and standard deviation across all directions.

## Discussion

OK requires accurate corneal topography to facilitate customised lens design and evaluation of treatment. This involves capturing data in a reproducible manner, that is not artefactually distorted and uses visually significant reference points. During videokeratoscopy, directing fixation to the centre of the Placido rings aligns the instrument axis with the visual axis, but displaces it from the pupillary axis and the corneal apex [36]. The resulting rotational misalignment of the cornea relative to the topographer can give rise to pseudo-keratoconus patterns in anatomically normal eyes. Angular decentrations as small as 5° can induce these maps [14, 36]. Studies also show that rotation systematically distorts topography symmetry indices [13]. Although these effects are well documented in healthy eyes, their impact on OK has not been examined. This study evaluated how rotational displacement between visual and geometric aligned scans alters OK maps in terms of treatment location and symmetry. The differences in treatment position observed between visual and geometric scans in the present study are predictable. The change in treatment position relative to the pupil can be modelled in terms of the pre-existing horizontal offset and the depth separation between these points (as shown in Eq. 1). Assuming a constant external anterior chamber depth (zP = 3.61 mm), the model closely reproduced measured shifts (bias ≈ 0.003 mm, *r* = 0.98) with small absolute errors. A cohort representative constant zP was used because subject-specific values were not available from other instruments. Individual variation in anterior chamber depth would be expected to influence the ΔxTP estimation. This simplification likely contributed to the mild proportional error at the most extreme shifts, and incorporating individual depth measurements would be expected to improve accuracy further. Across eyes, ΔxTP was strongly and inversely related to the extent of Placido shift (*r* = −0.93), consistent with a purely rotational effect of the topography coordinate frame. Furthermore, when symmetry metrics were referenced to the treatment centre, LR, SAI and SRI indices were indistinguishable between visual and geometric aligned maps. The practical message that follows is that rotational misalignment does not ‘corrupt’ the corneal data but rather shifts the topographic frame of reference.

OK also requires sufficiently stable reference points that are visually meaningful. In refractive-surgery modelling, the CSC and CSCLR appear to satisfy this prerequisite reasonably. For a typical cornea, a 10 D myopic correction centred on the CSC produces nasal shifts of approximately 0.10 and 0.015 mm for the CSCLR and CSC, respectively, after treatment [37]. Conversely, if treatment is centred on the CSCLR, then a temporal shift of approximately 0.015 mm occurs, while the CSC shows no shift [37]. OK typically treats much lower myopic powers, so the anticipated landmark shifts are likely to be even smaller. This study appears to support these modelling results as the absolute horizontal separation between the CSC and CSCLR (chord mu) was stable across treatment. The paired change was small and not significant (mean: −0.006 mm, 95% CI: −0.035 to 0.023). Nevertheless, chord mu is referenced to the pupil centre, which can shift with changes in diameter [20]. Accordingly, even with attempts to standardise illumination, between-visit differences in pupil size may introduce variability in chord mu. Rotation of the eye from visual to geometric alignment alters the position of the vertex normal, i.e., the topographic origin. This consequently displaces the position of the pupil and the OK treatment centre relative to this new vertex position. Given that visual alignment is required for the validity of the CSC and CSCLR reference points, any change in fixation by the participant during capture invalidates these references. Baseline corneal topography used for customised lens design should reflect these landmarks. Ideally, selected parameters need to encourage positioning towards the most visually optimal location on the cornea. Several studies suggest this to be the CSCLR, as it is the closest practical surrogate for the visual axis corneal intercept [15–17] and is not subject to change due to illumination. Despite this, centration on the CSC can yield similar optical outcomes [18, 19].

Currently, clinicians lack reliable methods of predicting overnight OK lens position. Because visual outcome is directly related to treatment location on the cornea, it is difficult to determine whether a particular lens design will be successful before initiating therapy. This study takes a step forward in addressing this issue through a MATLAB-based model, designed to predict OK lens decentration from corneal topography and lens sagittal height data. The model estimates the resting lens position by seeking the combination of horizontal/vertical translations and tilting that minimises asymmetry in simulated tear film clearance profiles. This approach contrasts with prior work that relies only on peripheral corneal data at fixed chord positions (often near 8 mm) [7, 22, 27]. Such data are particularly vulnerable to extrapolation errors, eyelid/eyelash artefacts and tear film instability, and critically do not account for dynamic lens–cornea interaction. In the framework described here, the clearance map updates as the lens is shifted and tilted, meaning prediction reflects the coupled interaction between lens geometry and corneal shape. The model’s agreement with clinical measurements was good for horizontal decentration (*r* = 0.77, *p* < 0.001, MAE 0.133 mm, Bland–Altman bias: −0.022 mm LoA: −0.355 to 0.311 mm). Deming regression suggested only modest proportional error (slope: 1.146, 95% CI: 0.709–1.582; intercept: −0.016 mm, 95% CI: −0.099 to 0.068). Performance deteriorated when central lens optic zone data were excluded from the optimisation procedure (*r* = 0.55, *p* = 0.004), indicating that central shape may provide meaningful constraints on where the lens settles. This challenges the common assumption that decentration is driven almost entirely by the lens peripheral zones. Vertical predictions were weaker (*r* = 0.23, *p* = 0.27, MAE 0.247 mm) with wider LoA, likely implicating factors that are not captured by corneal topography alone. This may include eyelid-related forces and potentially behavioural factors (e.g., sleep posture). Furthermore, modelling performance is contingent on the quality of the topographic data. The vertical meridian is more susceptible to extrapolation errors due to limited Placido ring coverage and potential interference from the lids and lashes, which may have reduced predictive accuracy.

Lens thickness and material density (specific gravity) are known to influence the position of daywear GP lenses [8]. These lenses also tend to be more stable with posterior displacement of the centre of gravity [38]. This could potentially be achieved in OK by increasing lens diameter and/or implementing a more oblate front surface geometry. Additionally, modulating lens thickness and front curvature distributions can influence lid-lens coupling, which can either dampen or accentuate lid influence. Adjustment of these parameters may help to optimise centration in OK. Since OK reshaping occurs overnight, the wearer’s sleeping patterns may also have the potential to affect outcomes. Limited study has been conducted in this area. Nevertheless, it has been shown that sleeping position can affect corneal staining patterns in dry eye and corneal asymmetry in keratoconus [39, 40]. During sleep, the eyes deviate upward after eyelid closure and remain elevated until REM onset, when they drop to a central or below central position, and demonstrate stereotyped, non-random movements in mostly oblique, less vertical and least horizontal configurations [41]. The effect of these eye movements on OK lens positioning is unclear, but it is unlikely to be insignificant. Incorporating eyelid-related biomechanics and other factors to improve vertical prediction is likely to be complex. Some variables may be partially captured using clinical proxies (e.g., palpebral aperture height/position, lid tension or observed lens behaviour during fitting), whereas others (e.g., sleep posture) would require external inputs such as questionnaires.

The location of the OK treatment position is dependent upon the topographic reference from which decentration is measured. Most studies examining OK centration report an inferotemporal bias and attribute this to the natural asymmetry of the cornea. With regard to the visual capture scans, this observation appears to hold true. However, it does appear illogical to assess corneal asymmetry from a position where the cornea is already tilted. By contrast, in the geometric scans, the overall temporal bias of treatment location disappears. This supports the findings of Douthwaite and Pardhan [42], who proposed that corneal tilt relative to the instrument axis accounts for the nasal-temporal asymmetry frequently observed in standard videokeratography. They hypothesised that if the tilt, measured with a rotationally symmetrical contact lens, closely matched the tilt of the natural cornea, then the cornea could also be considered rotationally symmetrical, with the observed asymmetry being an artefact caused by misalignment of the corneal apex. Their results supported this hypothesis, finding a statistically insignificant difference between natural corneal and contact lens tilt. The diminished temporal bias under geometric alignment observed in the present study may be further evidence that nasal-temporal asymmetry arises primarily about the CSCLR, and reflects a geometric bias introduced during visually aligned data acquisition rather than a true significant corneal asymmetry.

If one assumes that the cornea geometrically lacks significant quadrant asymmetry, then it follows that the inferior decentration bias observed in the current study must be reconciled with the other variables discussed earlier. Therefore, centration should be approached as a whole lens issue (front and back surface), rather than solely as a back surface or topography problem. This is where new trans-limbal OK designs have been applied to anecdotally provide improved lens positioning. These designs offer significantly larger diameters and engage the limbal-scleral shell, which is much more asymmetric than the cornea [43]. By positioning the lens underneath the eyelids, it can remain anchored in position, reducing the upper lid’s influence. These larger lenses also result in a posterior shift in the lens centre of gravity, which is advantageous in preventing decentration. Additionally, limbal or scleral asymmetries can be exploited through quadrant-specific peripheral lens zones. With respect to standard toric OK lenses, the effect on lens centration remains unclear. Batres et al. [31] concluded that there was no significant difference in treatment positioning between standard spherical and dual-axis corneal refractive therapy lenses. This is in contrast to an earlier study, which reported a reduced amount of decentration with toric OK lenses fitted on sufficiently astigmatic corneas [22]. The use of quadrant specificity and/or freeform customisation may be useful in exploiting the toricity of such corneas. Sun et al. [44] noted that topography-guided WAVE OK lenses (WAVE Contact Lens System, wavecontactlenses.com) produced less treatment decentration and better visual outcomes than a Euclid design. WAVE allows freeform OK lens profiles to be specified independently along eight semi-meridians (45° intervals), enabling a practical means to achieve a symmetric tear film clearance profile. Although the modelling in the present study was implemented through MATLAB and limited to spherical designs, the findings highlight tear film symmetry about an intended corneal landmark as an important design consideration. This can be achieved within custom platforms such as WAVE to promote more stable treatment localisation and improved visual outcomes. Future work should identify which design modifications most reliably improve lens positioning and how to incorporate external influences into prediction. Validation should also extend to a wider range of corneal astigmatism and refractive errors across multiple lens geometries, including toric, quadrant-specific and freeform designs.

HOAs are another important consideration when designing OK lenses. OK will induce HOAs irrespective of lens position, although they can become excessive depending on the lens design and amount of decentration [3, 29, 45]. Wang et al. [46] reported that temporal decentration >0.5 mm was associated with slower axial elongation than central treatment, with no significant association for other directions. In contrast, other work suggests that similar offsets appear to degrade visual quality without conferring additional myopia control benefit [5]. The model presented here demonstrates that for a 4.0 mm pupil, induced HOA RMS increased with decentration and accelerated beyond approximately 0.25–0.30 mm. As shown in Table 3, the effect of this was proportional to pupil size. A diameter of 4.0 mm appears to be a reasonable proxy for mean pupil size in young adults under photopic illumination [47]. Assuming a normal baseline HOA RMS of 0.10 µm at this pupil size [35], a doubling to ~0.20 µm would occur, on average, at ~0.40 mm decentration, providing a useful heuristic threshold. However, this is not a universal cut-off since induced aberrations varied with the direction of decentration in a subject-specific manner. The variability also increased with decentration magnitude and pupil size. Therefore, the effect of decentration should be interpreted individually in the context of pupil size, age, baseline HOAs, visual function and symptom tolerance. Currently, it is not recommended to decentre OK treatment deliberately for the purposes of myopia control, given the potential risk of adverse corneal effects such as staining, binding and warpage [48].

It is important to acknowledge that the HOA data in this study were estimated indirectly using corneal elevation Zernike modelling, rather than measured through wavefront aberrometry. Accordingly, visual symptoms and function (e.g., visual acuity, subjective refraction and contrast sensitivity) were not assessed. Incorporating true aberrometry into troubleshooting and pre-dispensing assessment can sharpen decisions when balancing intended optical effects for myopia control with decentration-induced HOAs. This retrospective study had limited standardisation of scan acquisition, sample size and repeat imaging. Analyses were performed at a single treatment time point (greater than 1 month) with variable follow-up intervals; therefore, temporal changes in centration could not be evaluated. Additionally, treatment zone shape metrics that may relate to decentration (e.g., ellipticity) were not analysed in this study. Future work will extend the analysis to include ellipticity, axis orientation and related metrics to determine if treatment zone geometry reflects decentration and whether this can be reproduced using lens/tear film modelling.

## Conclusions

The present study advocates for meaningful topography capture for OK treatment initiation and evaluation using visual scans. Differences between visual and geometric scans reflect predictable rotation of the topographic reference frame and do not appear to distort the appearance of OK treatment. Furthermore, the CSCLR appears to be a stable reference for designing lenses and for measuring treatment decentration. Topography data should be considered in conjunction with lens data to predict lens positioning in vivo. Considering the entire dynamic tear film profile, symmetry optimisation can produce useful predictions of horizontal decentration, whereas vertical prediction is weaker and likely driven by other factors such as eyelid forces. Induced HOAs increase with decentration and can vary by direction, so this should be considered when judging visual impact and determining alignment with treatment objectives. Lens designs should leverage any corneal asymmetry that exists in an attempt to encourage treatment to form at the most visually optimal location. This is generally considered to be at the CSCLR. Ideal vertical positioning of the lens may be more challenging to achieve in the absence of significant corneal asymmetry. In such cases, modulating non-corneal forces by selecting low-density materials, tailoring front surface design and adopting larger trans-limbal platforms may prove useful. Future research is needed to evaluate the effectiveness of these design choices.

## Data Availability

Data sets generated during the current study are available from the corresponding author on reasonable request.
